# Supplementary material for: Effects of Extracellular Vesicles from Osteogenic Differentiated Human BMSCs on Osteogenic and Adipogenic Differentiation Capacity of Naïve Human BMSCs
Source: Cells. 2022 Aug 11;11(16):2491. doi: 10.3390/cells11162491 (PMC9406723; doi:10.3390/cells11162491)
Supplement: Supplementary file 1 [file cells-11-02491-s001.zip › Cells-1841287-SM.pdf]

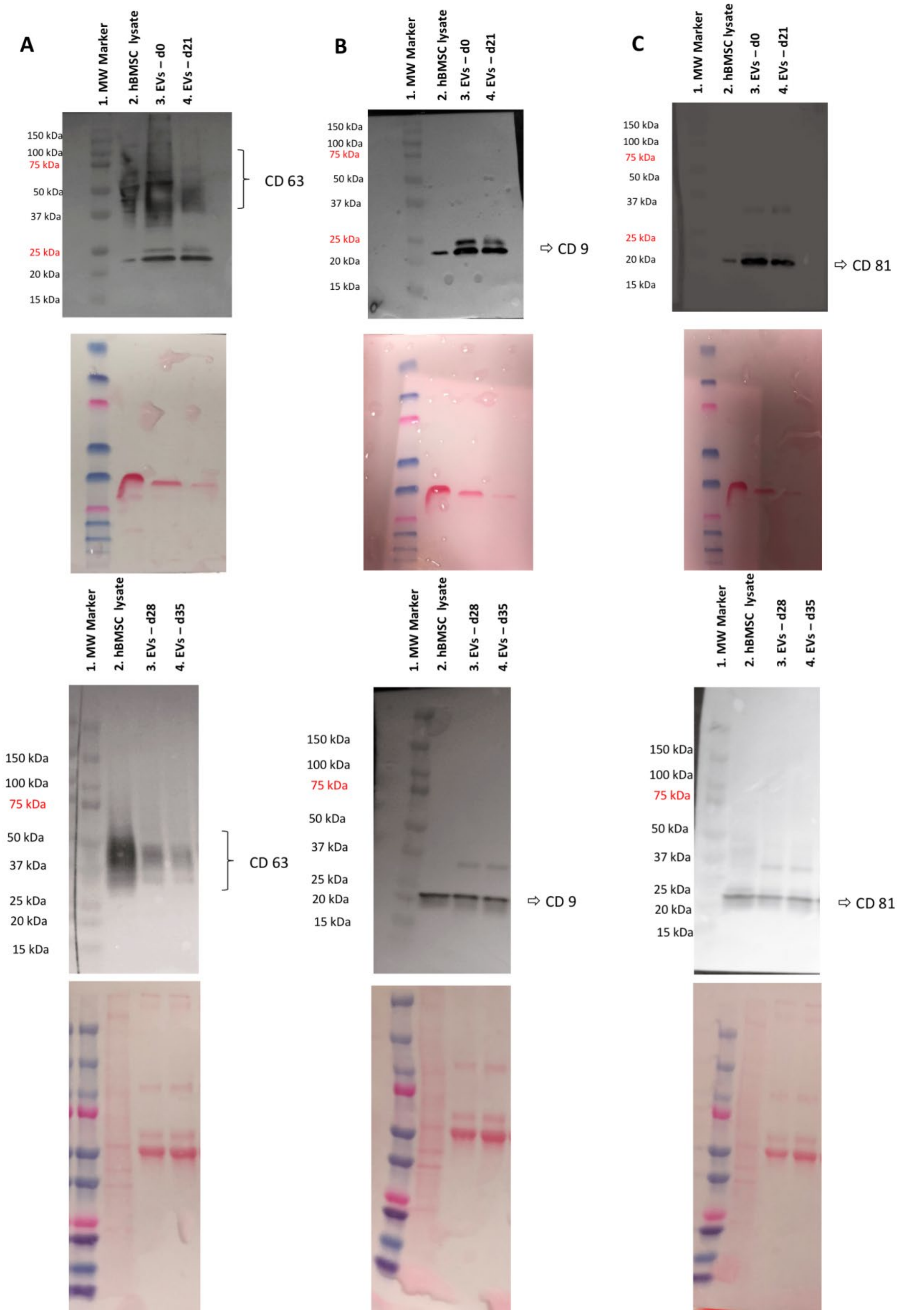

Suppl. figure S1. Western Blot analysis of classical surface markers of EVs

Western Blot analysis of EV surface marker proteins CD63 (A), CD9 (B), and CD81 (C) in hBMSC lysates (the top figure, lane 2, 30µg; the bottom figure, lane 2, 10µg), EVs_d0 lysate (the top figure, lane 3, 14µg), EVs _d21 lysate (the top figure, lane 4, 10.4 µg), EVs_d28 lysate (the bottom figure, lane 3, 10µg) and EVs_d35 lysate (the bottom figure, lane 4, 10µg); Lane 1 = MW ladder;

Respective Ponceau Red loading control images are shown below the respective blot membrane; n=2

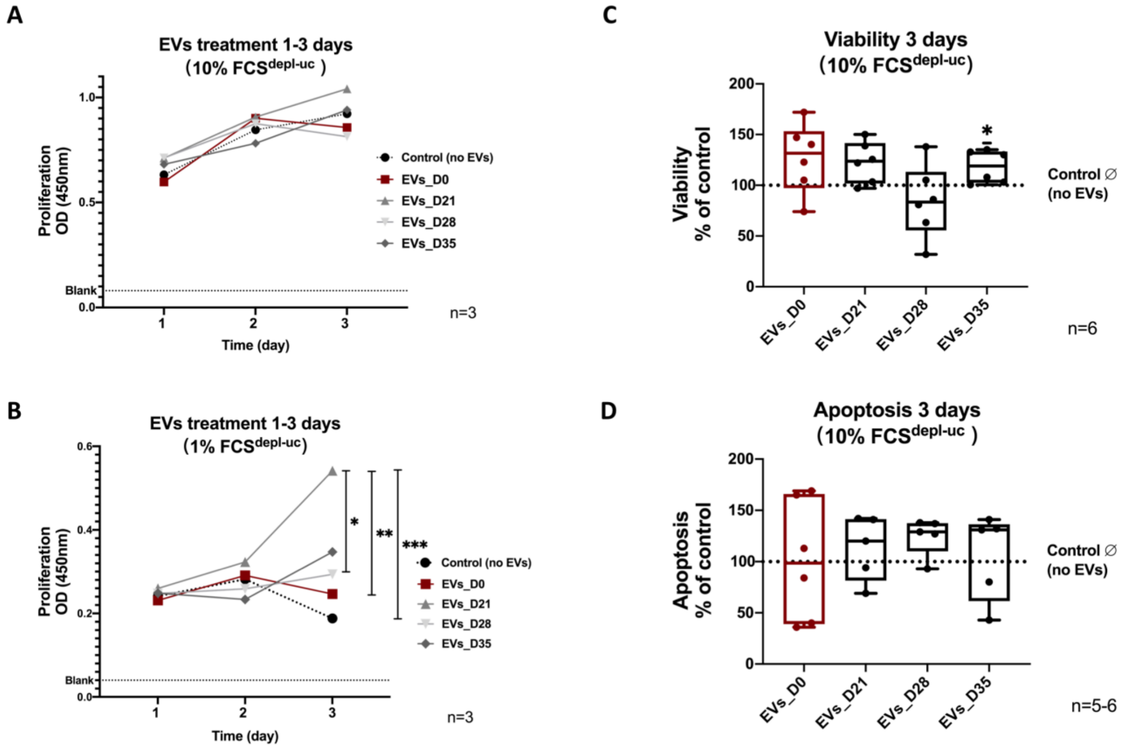

Suppl. figure S2. Effects of the different EVs groups on naïve hBMSCs cultured in the presence of 10% EVs-depleted FCS.

(A) Proliferation of naïve hBMSCs after stimulation with the different EVs groups or PBS (no EVs) in medium containing 10% EV-depleted FCS after 1, 2 or 3 days; n=3.

(B) Analysis of proliferation of naïve hBMSCs after stimulation with the different EV groups or with PBS (no EVs) in medium containing 1% EV-depleted FCS for 1, 2 or 3 days. *$p < 0.05$; **$p < 0.01$; ***$p < 0.001$; Two-way ANOVA with Tukey's multiple comparisons test; n=3.

(C) Viability and (D) apoptosis of naïve hBMSCs after stimulation for 3 days with the different EVs groups or PBS (no EVs) in medium containing 10% EV-depleted FCS. Results were calculated as percentage to the control group (no EVs, shown by the dotted line); *Compared with no EVs group, *$p < 0.05$; Wilcoxon signed-rank test was used when no EVs group was set to 100%, and two-tailed Mann-Whitney U-test was used to compare differences between different EVs treated groups. n=5-6.
